# HylleraasMD: A Domain Decomposition-Based Hybrid Particle-Field Software for Multiscale Simulations of Soft Matter

**DOI:** 10.1021/acs.jctc.3c00134

**Published:** 2023-05-02

**Authors:** Morten Ledum, Samiran Sen, Xinmeng Li, Manuel Carrer, Yu Feng, Michele Cascella, Sigbjørn Løland Bore

**Affiliations:** † Department of Chemistry and Hylleraas Centre for Quantum Molecular Sciences, University of Oslo, PO Box 1033 Blindern, 0315 Oslo, Norway; ‡ Berkeley Center for Cosmological Physics and Department of Physics, University of California, Berkeley, California 94720, United States; § Department of Chemistry and Biochemistry, University of California San Diego, La Jolla, California 92093, United States

## Abstract

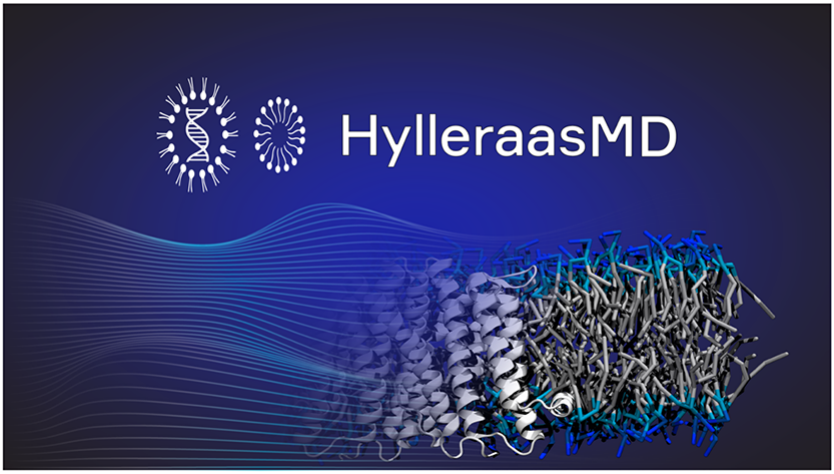

We present HylleraasMD (HyMD), a comprehensive implementation of the recently proposed Hamiltonian formulation of hybrid particle-field molecular dynamics. The methodology is based on a tunable, grid-independent length-scale of coarse graining, obtained by filtering particle densities in reciprocal space. This enables systematic convergence of energies and forces by grid refinement, also eliminating nonphysical force aliasing. Separating the time integration of fast modes associated with internal molecular motion from slow modes associated with their density fields, we enable the first time-reversible, energy-conserving hybrid particle-field simulations. HyMD comprises the optional use of explicit electrostatics, which, in this formalism, corresponds to the long-range potential in particle-mesh Ewald. We demonstrate the ability of HyMD to perform simulations in the microcanonical and canonical ensembles with a series of test cases, comprising lipid bilayers and vesicles, surfactant micelles, and polypeptide chains, comparing our results to established literature. An on-the-fly increase of the characteristic coarse-grain length significantly speeds up dynamics, accelerating self-diffusion and leading to expedited aggregation. Exploiting this acceleration, we find that the time scales involved in the self-assembly of polymeric structures can lie in the tens to hundreds of picoseconds instead of the multimicrosecond regime observed with comparable coarse-grained models.

## 1 Introduction

Hybrid particle-field simulations (hPF) are computationally efficient approaches for studying mesoscale soft matter systems with molecular resolution.^1−5^ In hPF models, intermolecular pair interaction potentials are replaced by particle-field interactions that functionally depend on particle densities. The low computational cost of particle-field interactions and their soft nature make them efficient for sampling equilibrium statistics of challenging systems involving kinetic traps, molecular entanglement, and crowding.

Starting from early density-field models where mesoscopic densities in condensed systems were optimized by self-consistent procedures, and through pioneering hybrid models by Zuckermann coupling particles through density fields,^6^ hPF models have reached maturity through particle-mesh implementations (PM) with a sampling of the conformational space either by Monte Carlo *single chain in mean field*(1,2,7) or by molecular dynamics (MD).^3,4^ Successful examples of the methodology span from polymer melts,^3,8,9^ lamellar and nonlamellar phases of lipids and surfactants,^10−12^ percolation properties of nanoparticles and carbon nanotubes^13,14^ to charged surfactants and polypeptides.^15−18^

Recently, two of us presented a new Hamiltonian formulation for the hPF-MD approach (HhPF), where the microscopic forces acting on the particles are directly obtained by the spatial derivative of the interaction energy functional.^19^ Importantly, the level of coarsening in hPF methods is determined by the density spread associated with the molecular moieties. Such spread is commonly defined by adopting coarse grids on which particle-mesh operations are defined.^3,4^ In the new HhPF formalism, we decouple the density spread from the grid refinement by employing filtered densities with an intrinsic filtering scale. This procedure, similar to the Gaussian spread of point charges in the Ewald method,^20^ decouples the model’s resolution from the operations associated with evaluating the density and density gradients, thus allowing for systematic numerical convergence of the hPF forces.^19^ In particular, testing HhPF on ideal monatomic systems, it was possible to demonstrate a systematic reduction of aliasing as well as excellent conservation of energy by increasing the number of mesh points.^19^

Given the apparent advantages of the filtered formulation of hPF simulations, it is of great interest to further pursue this approach beyond toy systems to realistic molecular assemblies at the mesoscale. A proper analysis of the HhPF framework as applied to realistic soft matter systems necessitates an implementation beyond the preliminary code presented in ref (19). Specifically, coarse grained simulations of macromolecules require intramolecular bending, stretching, and torsional potentials. Moreover, explicit handling of long-range electrostatic forces may be needed for a range of biologically important molecules, such as charged lipids, proteins or long polyanionic nucleic acids. Specific to hPF modeling of peptides,^18^ we implement topological reconstruction of permanent dipoles, which has been shown to reproduce all-atom electrostatic forces.^21^

Uncoupling the spatial evaluation of the densities from the computational grid allows for an arbitrary definition of the density spread, which acts as the coarse graining parameter.^19^ Here we check the effect of the particle spread on the dynamic behavior of test molecular systems. In particular, we explore the possibility of tuning the density spread *on-the-fly* to significantly accelerate the aggregation dynamics of self-assembling systems.

A big advantage of hybrid particle-field models is the sped-up dynamics of collective processes of supramolecular structures, such as the self-assembly of biological lipids. The fast aggregation is partly due to the intrinsic softness of the hPF potential and partly due to the coarse-grained representation of the molecules. Through tuning of the latter by varying the spread of the grid-independent window function, even further speed-up of aggregation dynamics is achieved. We demonstrate ultrafast self-assembly processes for large filtering scales beyond comparable coarse-grained simulations previously reported. This enables us, in principle, to probe aggregation of molecular structures not normally accessible in hPF-MD frameworks.

The current state-of-the-art parallelization approach for hPF simulations, including implementations by Müller or Milano,^22,23^ and the GPU-based Galamost code^24,25^ is *the shared memory strategy*. In this strategy, molecules are permanently assigned to MPI-tasks, and all MPI-tasks share the whole density-field grid. Communication is only needed when combining the densities from the different MPI-tasks. In this regime, the combination of using a low spatial resolution representation of the grid, and infrequent updates, has allowed applications with excellent scaling behavior demonstrated in hPF benchmark studies.^22,23^

The HhPF approach requires a higher number of grid points in order to achieve increased accuracy and better numerical control over the hybrid particle-field dynamics. The shared memory strategy is not well suited for an efficient implementation in the new framework because the serial computational costs associated with the grid computation quickly become the bottleneck. For the current implementation, we opted for a *domain-decomposition strategy*, in which the grid operations are performed jointly by all processors handling individual subsets of the entire simulation box.

In the following, we validate the HhPF formulation for realistic molecular systems, using selected soft matter systems as test cases. We demonstrate the HhPF scheme’s ability to accurately model the aggregation and equilibrium structures of lamellar and nonlamellar phospholipid phases, charged lipids, charged organic surfactants, and model peptides. We also benchmark the first full implementation of a HhPF-MD code. We name the code presented here *Hylleraas MD* (HyMD hereafter), after the Hylleraas Centre for Quantum Molecular Sciences, where the HhPF approach has been first formulated and developed.

## 2 Theory and Methods

### 2.1 Hamiltonian Hybrid Particle-Field

In HhPF, we consider a system of *N* interacting particles in *M* molecules (each containing *N*_*m*_ particles) at positions  with conjugate momenta . The subsets of position and momentum vectors contained in molecule *m* are labeled **r**^*m*^ and **p**^*m*^. The particles are subjected to the Hamiltonian:
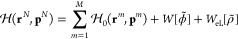
1Here,  is the Hamiltonian of a single noninteracting molecule *m*, and  is an interaction energy functional depending on the filtered particle number densities :
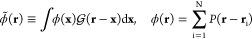
2where  is a filter function, and *P* is a window function used to distribute the particles in the space. The  term denotes the electrostatic interaction energy functional, depending on the filtered charge density, , and the particle charges, *q*_*i*_:
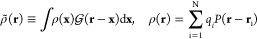
3

The sampling of the phase space associated with eq 1 using MD requires computing the forces due to , *W*, and *W*_el._. The forces due to bonded interactions terms of single molecules (denoted *U*(**r**^*m*^)) are computed by
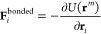
4The bonded potentials used in the present work are standard terms used in many other all-atom and coarse-grained force fields. A short introduction to the specific terms is included in the Supporting Information (section S4). The forces due to particle-field interactions, in the presence of a local energy functional of the form: , are obtained as^19^
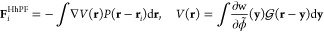
5where *V* is the external potential acting on the particles. Since the implementation of the bonded forces is no different from any other MD software (see ref (26)), we only describe how the HhPF forces are computed.

#### 2.1.1 Computation of Density on a Grid

The estimation of discrete densities is done using a cloud-in-cell (CIC) window function *P*, which distributes particles on the nearest grid points by trilinear interpolation. The density is computed at grid point  by
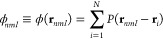
6where  is the position of the grid vertex with indices . Note that the window function *P* centered on grid-point  will normally vanish on all but the closest grid points, *n* ± 1, *m* ± 1,  ± 1.

#### 2.1.2 Determination of the External Potential

Considering functionals locally dependent on , the first step is to obtain . A straightforward way of obtaining it is by Fast Fourier Transform (FFT):
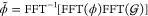
7where we have used the convolution theorem. Next, we find the external potential as
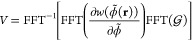
8The derivative of *V* is computed in Fourier space:
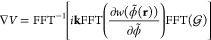
9

#### 2.1.3 Force Interpolation

The forces are computed by interpolating back the derivative of the external potential from the grid vertices onto the particles through eq 5 by
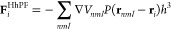
10where *h*^3^ is the volume of a single grid cell. Note that once again, the sum is taken over *all* grid-point triplets , however *P* will normally vanish on all but the closest few grid points around the position of particle *i*, **r**_*i*_.

#### 2.1.4 Interaction Energy Functional

As a model for intermolecular interactions we consider the standard energy mixing potential commonly adopted in hPF-MD^3^ and SCMF,^1^ this time defined using filtered densities:
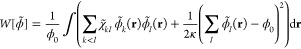
11where  is the Flory–Huggins mixing parameter between particle species *k* and , κ is a compressibility parameter and ϕ_0_ is the average density of the system. The corresponding external potential is given by
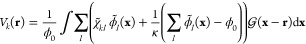
12The full specification of the model requires defining , the grid independent window function. Following ref (19), we implemented a Gaussian filter:
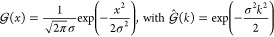
13where the standard deviation σ is an indication of the space occupied by the particle, that is, the level of coarsening by the density representation.

The formalism described here is entirely Hamiltonian-agnostic; likewise is the developed HyMD code, where symbolic differentiation in the SymPy library^27^ is used to obtain forces derived from *any* Hamiltonian functional form (local or otherwise). Additionally, this also holds for the window function, any function specified as a filter may be used and is automatically handled by the software.

### 2.2 Electrostatic hPF Interactions

In the usual Ewald formulation, a set of point charges are screened with Gaussian charge distributions giving rise to a short-range electrostatic interaction. The addition of the compensating Gaussian charges yields a smoothly varying charge density, producing the long-range part of the electrostatics. Unlike in standard hPF implementations,^15,28^ within the HhPF formalism, the particle beads are *intrinsically* smeared, filtered density distributions. In the case of a Gaussian window function, this gives rise to only a long-range part of interacting screening charges akin to the long-range part of the Ewald summation. Circumventing the short-range part altogether enables us to compute the electrostatic potential and electric field entirely in reciprocal space. In terms of the filtered *charge* densities , we obtain the grid charge densities via
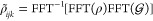
14and the electrostatic potential Ψ_*ijk*_ as
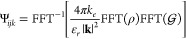
15where *k*_*e*_ denotes the Coulomb constant *k*_*e*_ = 1/4*πε*_0_, and ε_*r*_ denotes the relative permittivity. The electric field is found through
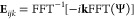
16with the forces obtained by trilinear interpolation of the electric field from grid values back onto particle positions:
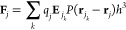
17

### 2.3 Angular-Torsional Potential and Dipole Reconstruction for Peptide Simulations

Following up on recent hPF developments for the simulation of peptide chains,^18^ in HyMD we implement a combined bending-torsional potential to describe the mechanics of the backbone atoms of polypeptides:
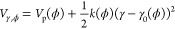
18where *k*(ϕ) and *V*_p_(ϕ) both are represented by cosine series of the dihedral angle and γ_0_(ϕ) adapted from ref (29). The *propensity* potential *V*_p_(ϕ) determines the presence and the relative energy of any minima along ϕ, while *k*(ϕ) governs the strength of the harmonic deviations of the bending angle γ from the ideal γ_0_(ϕ) value.

As shown in ref (21), from the positions of the C_α_s along the peptide backbone, it is possible to topologically reconstruct dipoles mimicking the presence of peptide–peptide interactions. In the simulation the dipoles are represented as a pair of ghost charges of strength ± *q* located at
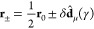
19where **r**_0_ is the C_α_–C_α_ position vector, δ is the half distance between the dipole charges, and the unit vector  is the direction of the dipole moment, which depends on the angle γ between triplets of successive C_α_s.^21,30^ The electrostatic forces acting on the dipoles are then projected onto the backbone atoms so that the charge positions do not have to be propagated with MD.^18,30^

### 2.4 Implementation strategy

#### 2.4.1 Parallelization Strategy

**Figure 1:**
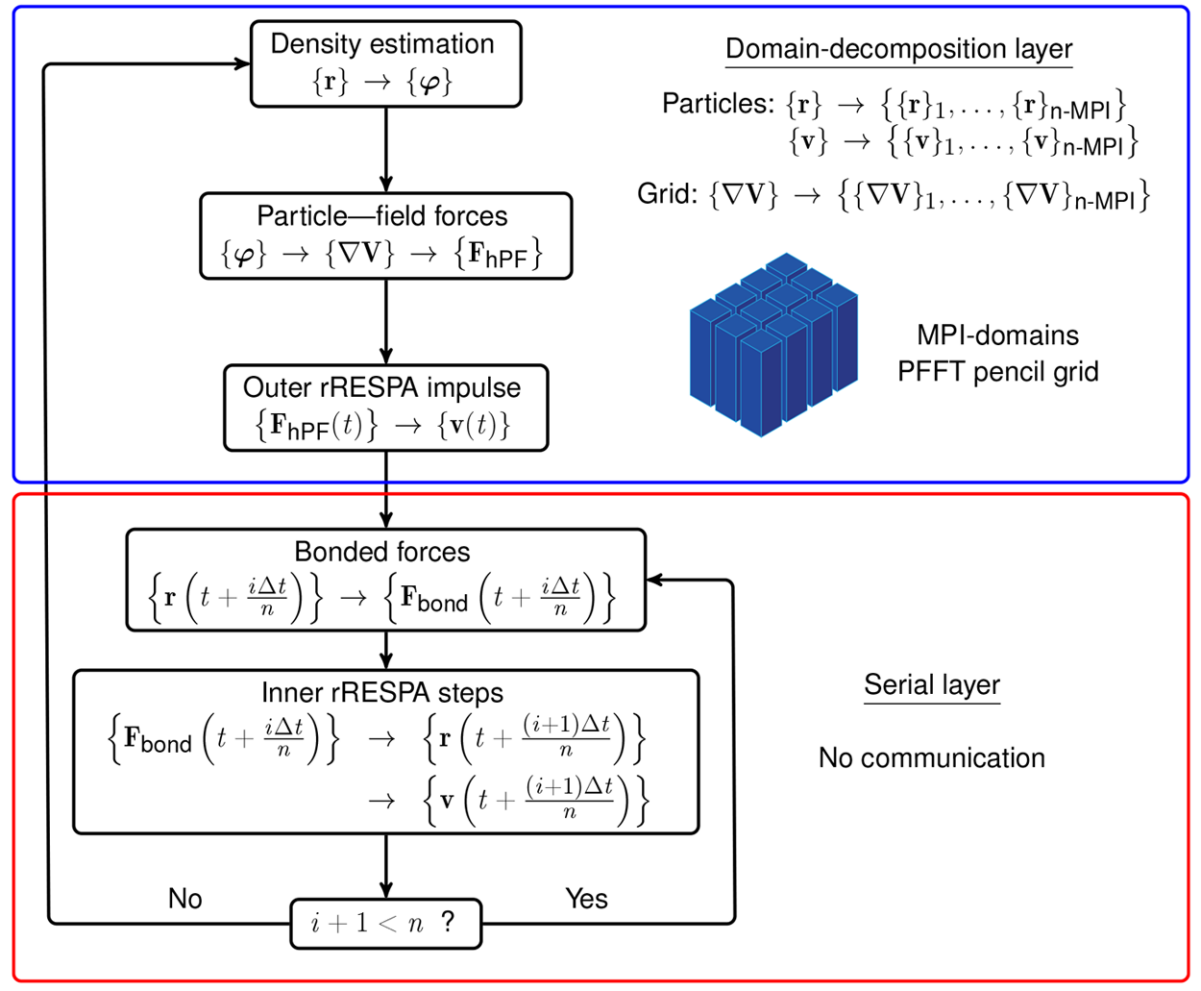
Simulation protocol using the reversible-RESPA integrator with a domain-decomposition parallelization strategy. Domain-decomposition is typically done every hundred of thousands of time steps. During integration, the estimation of the density field and the computation of particle-field forces is the only part of the algorithm which requires internode MPI communication. The inner rRESPA steps, typically done tens to hundreds of times for each field update, are entirely serial and embarrassingly parallel in nature.

Parallelization of the computational operations involved in hPF-MD is essential to model systems at experimentally relevant length and time scales. On the one hand, the most costly operations, including grid operations, most notably FFT and bonded forces, need to be parallelized. On the other hand, the overhead associated with the parallelization, which impairs the performance, must be reduced to a minimum. Our parallelization approach exploits simplifications that are provided by a multiple time step algorithm to satisfy both aspects. Specifically, we have two layers (see Figure 1). In the domain-decomposition layer, we divide the particles and the density-grid into MPI-domains in a pencil grid arrangement according to their spatial location.^31^ This provides scalability for large systems while reducing communication, by minimizing the amount of data transferred between MPI domains after each 1D Fourier transform. This layer computes particle-field forces and assigns particles to MPI tasks. Next, we have a serial layer. This layer computes bonded forces and integrates the equations of motion. Since the particle-field forces are constant in this layer, there is no communication between processors. This layer thus exhibits excellent scaling behavior.

#### 2.4.2 Multiple Time Step Algorithm

We implement a reversible reference system propagator algorithm (rRESPA) integrator.^32^ Starting from the decomposed Liouville operator in two parts *iL* = *iL*_1_ + *iL*_2_, the Trotter factorization^33^ gives the classical time propagator:
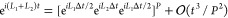
20where Δ*t* = *t*/*P*. The resulting discrete time propagator takes the form:
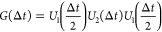
21which is unitary and hence time reversible by virtue of *U*_1_ and *U*_2_ being individually unitary. Considering a clever factorization of the full Liouville operator into a *reference system* of intramolecular forces *F*_M_, and a part which describes the deviation of the reference system from the full system by the field forces *F*_F_. With  being the intramolecular Liouville operator, the full Liouvillian takes the form:
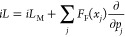
22which becomes
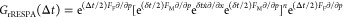
23by application of the Trotter factorization. Eq 23 dictates integration of the intramolecular forces by velocity-Verlet in increments of the *inner* time step *δt* = Δ*t*/*n*, *n* times. The slowly varying field forces are applied as infrequent impulses once per full time step of length Δ*t*. As the field force impulse only corrects the *velocities* of the reference system, the force is unchanged at the start of the subsequent time step and no recomputation of the field force is necessary for the initial pulse at the start of the next step. The implemented rRESPA algorithm is presented in Figure 1.

In total, *n* calculations of the intramolecular forces *F*_M_ are required per Δ*t* of integration, in addition to one computation of *F*_F_. In the limit of *n* = 1, the rRESPA integrator becomes a simple velocity-Verlet integrator.

### 2.5 Implementation Details

The *HyMD* HhPF code is the expansion of an early implementation developed in ref (19). HyMD is written almost purely in python with MPI parallelization through the mpi4py library.^34^ Its key functionality is implemented as follows. The PM computations needed to compute particle-field forces are done by using the PMESH library.^35^ The PMESH library has MPI parallelized routines for interpolating density and forces. The most costly operation, the FFT, is computed by the highly scalable PFFT package tailored for dealing with huge grids.^31^ MD trajectories and energy information is stored using the H5MD format,^36^ based on the HDF5 file format,^37^ using the python package h5py^38^ with the MPI driver. This file format enables efficient parallel input/output for production runs in application studies involving large amounts of data. Finally, the rRESPA integrator, bonded forces (including stretching, bending, and dihedral potentials), electrostatic interactions, and canonical sampling by velocity rescale (CSVR) thermostat^39^ have been implemented.

HyMD is publicly available under a GNU Lesser General Public License v3.0 (LGPLv3) at our GitHub Web site https://github.com/Cascella-Group-UiO/HyMD. The LGPLv3 open source software license allows anyone to freely use and modify the software, as long as the changed code is also made freely available under an equivalent license.

### 2.6 Simulation Details

We consider as a prototypic test case the coarse-grained dipalmitoylphosphatidylcholine (DPPC) lipid model. In addition to this fully satured phospholipid, we also use a monounsaturated dioleoylphosphatidylcholine (DOPC) lipid, along with a short model polypeptide consisting of single bead alanine (ALA) amino acids that is hydrophobic in the core and hydrophilic in the ends. To test the implementation of hPF electrostatics, we further consider a coarse-grained Lipid A model. The phospholipid systems use parametrizations previously reported by us,^40^ while the lipid A parameters are developed by De Nicola et al.^41^ In both cases, the four-to-one heavy atom MARTINI^42,43^ mapping with explicit solvent is used. Finally, we test the aggregation of charged 4-butyl-4-(3 trimethylammoniumpropoxy)-phenylazobenzene (AzoTMA) surfactant using a finer two-to-one heavy atom mapping (also with explicit solvent) to account for the ring structures. The mapping is based on ref (44) (Figure S1, in Supporting Information), and  parameters were developed by us. All interaction energy parameters used in the present work are presented in Table S1 in Supporting Information.

The incompressibility parameter is fixed at κ^–1^ = 7.45*RT*, in correspondence with what has previously been reported to reproduce particle–particle CG density fluctuations.^11^ Whenever the canonical ensemble is sampled, a CSVR thermostat with characteristic coupling time 0.1 ps is used, and unless otherwise noted, the time step of the inner rRESPA steps (bonded forces) is 0.01 ps in accordance with the stability criterion of the intramolecular forces, as previously demonstrated by Wigner et al.^45,46^Table 1 shows the composition of all systems simulated. The phospholipid systems are all generated using CHARMM-GUI,^47−49^ and MARTINI simulations as well as CHARMM36^50,51^ all-atom simulations are performed with the Gromacs^52^ software package.

**Table 1:** Overview of Simulated Systems

System	Molecules	Method	Solvent^a^	Counterions	Box size [nm^3^] (*x*/*y*, *z*)	Ensemble
DPPC1	318 DPPC	All-atom-PME^b^	19711	–	10.0^3^^d^	NPT
DPPC2	318 DPPC	All-atom-PME^b^	19711	–	9.83^2^ × 10.15	NVT
DPPC3	318 DPPC	MARTINI-RF^c^	4927	–	9.83^2^ × 10.15	NVT
DPPC4	318 DPPC	HhPF	4927	–	9.83^2^ × 10.15	NVT
DPPC5	1272 DPPC	HhPF	50757	–	20.04^2^ × 19.39	NVE/NVT
LIPIDA	644 Lipid A	HhPF-PME	211555	644 Ca^2+^	30.0^3^	NVT
AZOTMA1	90 AzoTMA	MARTINI-RF^c^	5833	90 Cl^–^	9.0^3^^d^	NPT
AZOTMA2	90 AzoTMA	HhPF-PME	5833	90 Cl^–^	9.0^3^	NVT
PEPTIDE	1148 DOPC, 30 ALA	HhPF-PME	51159	–	19.70^2^ × 19.46	NVT
MELT1	22499 HP^e^	HhPF	–	–	30^3^	NVT
MELT2	53331 HP^e^	HhPF	–	–	40^3^	NVT
MELT3	104162 HP^e^	HhPF	–	–	50^3^	NVT

a Solvent denotes coarse grained four-to-one waters, except for DPPC1 and DPPC2 for which TIP3P water is used.

b All-atom simulations performed using the CHARMM36^50,51^ force field.

c MARTINI simulations use reaction field (RF) electrostatics.

d Starting simulation box size, prior to constant pressure equilibration.

e MELT*N* systems contain homopolymers (HP) of length 10.

An overview of the different systems simulated in this work is presented in Table 1. All phospholipid systems are first equilibrated under constant pressure conditions in either all-atom or CG (MARTINI) simulations. The box size is then averaged over several tens of nanoseconds and fixed for use in constant volume simulations in HyMD. Constant pressure sampling has been recently introduced within the hPF-MD formalism.^53^ Its further development and implementation within the new HhPF framework has been recently presented.^86^

## 3 Results and Discussion

### 3.1 Conservation of Energy and Center of Mass Momentum

**Figure 2:**
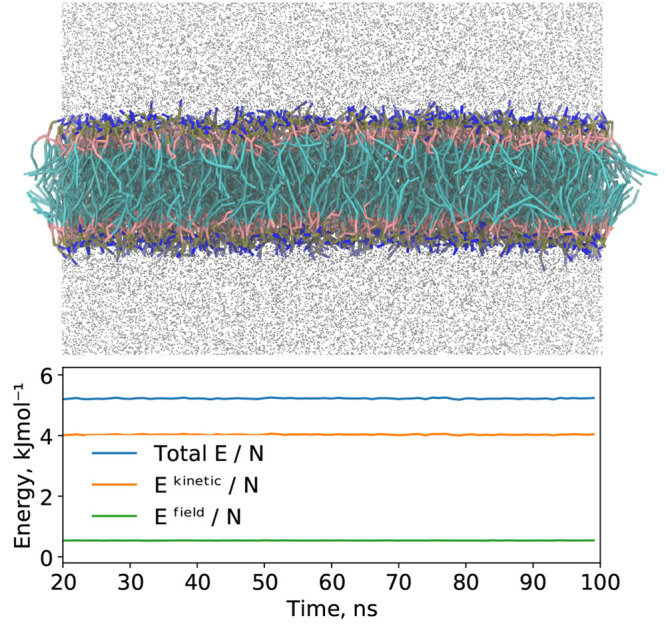
Representative snapshot from a HhPF simulation, along with the energy per particle (total, kinetic, field) for a DPPC bilayer test system simulated under constant energy conditions with solvent water beads shown in silver (DPPC5 system, see Table 1). A fine grid spacing of σ/*h* = 4.7 was used, with an equilibration period of 20 ns before the sampling was started. The relative drift in the total energy per ns simulated was 0.0015%.

We report the first ever constant energy hybrid particle-field simulation of a solvated phospholipid system. As validation of the implementation of HyMD, we present in Figure 2 the energy of the DPPC5 system (Table 1). During HhPF simulations the energy is well conserved, with an average relative drift of 0.0015% per nanosecond. Likewise, the center of mass momentum accumulates 0.024 amu m s^–1^ per nanosecond per particle (Figure S2, in Supporting Information). As with any MD approach, the level of energy and momentum conservation is determined by the time step and floating-point precision used. As the main computational load of the HyMD program is due to the FFTs, it is prudent to employ single-precision floating point numbers to represent positions and velocities. This choice yields marginally worse conservation of energy, but has no apparent effect on the conservation of total momentum. As previously noted by us,^19^ the degree of energy conservation is in large part also determined by the level of coarse-graining, σ, and the grid spacing, *h*. A larger ratio of σ/*h* yields more stable energies and momenta.

### 3.2 Multiple Time Step Integration

When using coarse grids (many particles per grid cell) or filtering on coarse length scales, the external potential is slowly evolving compared to the internal motions within molecules, such as stretching and bending motions. This difference in time scales has been algorithmically exploited in both the Monte Carlo-based SCMF^2^ and in the MD-based formulation,^3,4^ applying the quasi-instantaneous approximation, where the external potential is kept constant for multiple time steps. We recently demonstrated that just using larger time steps in the integration of the field forces yields superior conservation of energy, while also avoiding unphysical production of net momenta.^19^ In contrast to the quasi-instantaneous approximation used in previous formulations of hybrid particle-field, the reversible reference frame integration algorithm yields time reversible equations of motion integration, giving favorable integration accuracy and increased stability.

**Figure 3:**
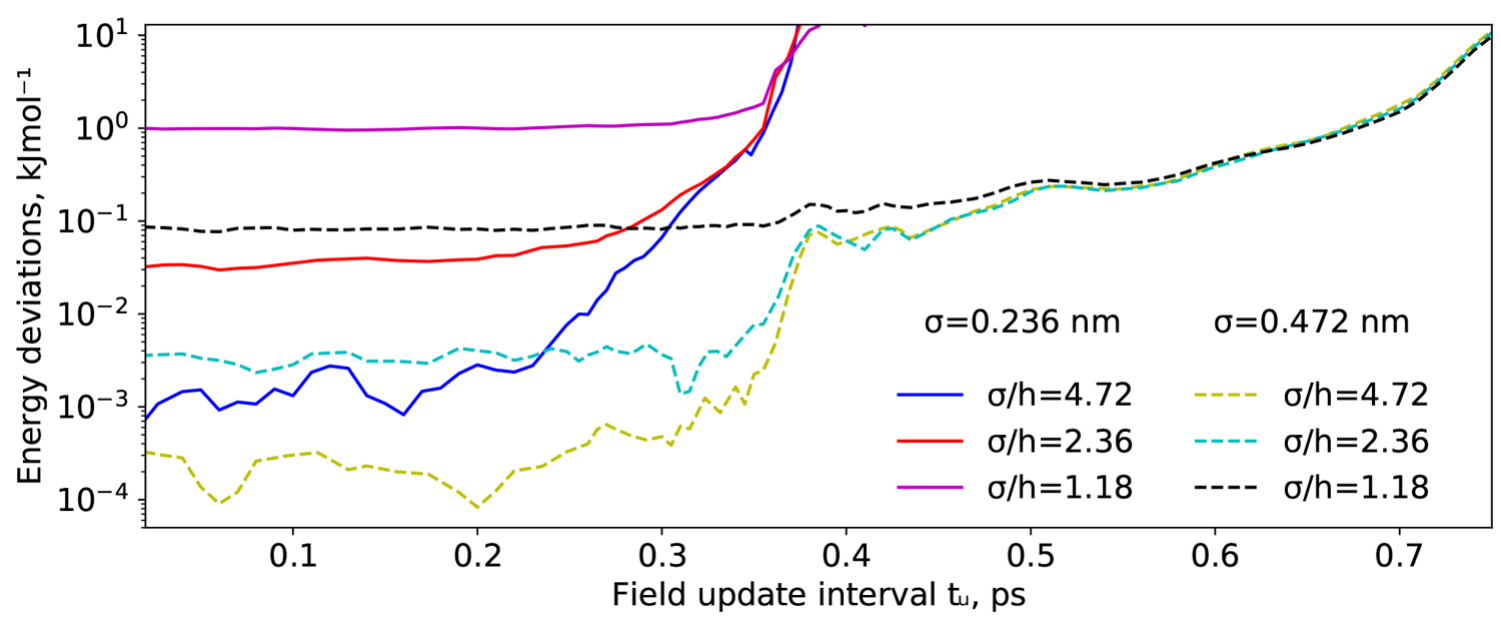
Absolute energy drift per particle per nanosecond for a DPPC bilayer test system (DPPC4, see Table 1) under constant energy conditions as a function of the update interval of the field forces, *t*_u_. Full lines represent simulations at the hPF-matching coarse-graining level σ = 0.236 nm, while dotted lines represent σ = 0.472 nm simulations.

Increasing the number of intermediate rRESPA steps has an almost linear impact on simulation speed-up because of the embarrassingly parallel nature of the intramolecular force calculation. For efficiency it is thus important to use the maximum allowable number of inner integrator steps. In order to gauge how long the inner rRESPA step can be, without decreasing the quality of the microscopic mechanics, we report in Figure 3 the energy conservation of the DPPC test system for varying values of the field update time, *t*_u_. Larger σs give rise to smoother and more slowly varying density fields, allowing longer update intervals before the scheme breaks down. This is exemplified at σ = 0.236 nm, with the necessary update time being around 0.25 ps, while in the more coarse case of σ = 0.472 nm, a longer interval of approximately 0.3 ps is acceptable. In each case of σ, the region in which the energy conservation breaks down is approximately unchanged for the different grid spacings, h, used. Thus a higher ratio of the coarse-graining parameter to the HhPF grid spacing yields better overall energy conservation, but does not appear to have a big impact on the stability of the energy with respect to *t*_u_.

### 3.3 Hamiltonian hPF-MD Simulations of Phospholipid Bilayers

**Figure 4:**
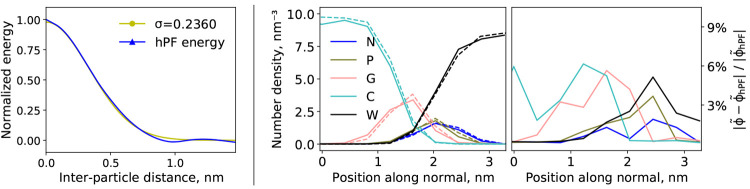
Left: Field-potential energy in standard hPF (blue) compared to grid-converged HhPF (yellow) for varying interparticle distances in a simple two-particle system at the best fit σ. Right: Symmetrized partial density profiles for unfiltered hPF (full lines) and HhPF with σ = 0.236 nm (dotted lines) NVT simulations of solvated DPPC membranes at 323 K (DPPC5, see Table 1). The absolute relative error is shown on the right.

An approximate equivalence may be established between the smoothed-out density approach in the HhPF framework with a Gaussian filter and standard hPF-MD formulation.^3,4^ Calibrating the grid independent window function width σ to match hPF forces and energies (at a standard grid length of 0.5875 nm), yields the best match value at σ_0_ = 0.236 nm ±0.00098 nm. This is illustrated in Figure 4, where the potential energy of a simple two-particle system in both frameworks is shown. Note that the fitting is done using a grid-converged HhPF, i.e., a Gaussian core model.^19^ Using this value of the window function width, σ_0_ ≡ 0.236 nm, reoptimization of the interaction energy parameters  may be circumvented, while still retaining the structural properties of the system under canonical hPF.

#### 3.3.1 Transferability of Interaction Parameters

While energy conservation provides an internal validation of both the approach and the software implementation, it does not provide external measurement of the quality of the model. Thus, we verify the transferability of standard hPF  parameters from literature data^40^ to the HhPF formulation by constant temperature simulations of the same DPPC bilayer system. Figure 4 shows comparisons of lateral number density profiles for DPPC membranes for unfiltered hPF and HhPF MD. Employing the target σ_0_ value, the HhPF framework satisfactorily reproduces the structures found with hPF, with a relative difference (relative to the total number density) of no more than 6%. This result indicates that literature hPF parameters are highly transferable to HhPF simulations, provided σ is adequately calibrated.

#### 3.3.2 Comparison of HhPF Structure and That of All-Atom and MARTINI

**Figure 5:**
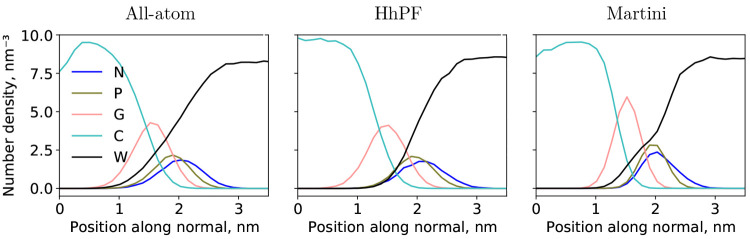
Symmetrized partial density profiles for all-atom (left), HhPF with σ = σ_0_ (this work, middle), and MARTINI^43,54^ (right) NVT simulations of solvated DPPC membranes at 323 K (DPPC1, DPPC3, and DPPC5 systems, respectively, see Table 1). The all-atom trajectory was coarse-grained with the four-to-one heavy atom MARTINI mapping before the profile was calculated. The HhPF (middle) and MARTINI simulations were both run using the same coarse-graining level. In each case, an equilibration time of at least 20 ns was allowed before sampling for at least 80 ns.

In order to further assess the quality of the HhPF bilayer system using the hPF-like σ = σ_0_, we compare it to all-atom (CHARMM36^50,51^ force field) and coarse-grained MARTINI structures. Figure 5 presents partial density profiles of the three cases. The MARTINI model appears too *stiff* to capture the small wavelength undulations present in the all-atom simulation. Such small fluctuations smooth out the calculated profiles when averaging the coarse-grained representation over many trajectory steps. The intrinsically softer HhPF model is better able to capture this flexibility of the membrane structure, yielding overall very satisfactory agreement between the all-atom and HhPF densities of lipid heads and glycerol groups (N, P, and G coarse-grained beads). The major discrepancy in the HhPF model is related to less water penetration into the lipid bilayer, likely due to a too high  value between the carbon and water beads. The better agreement of HhPF to all-atom data than the MARTINI is surprising, especially because these hPF parameters were originally optimized with respect to MARTINI data.^40^ This effect is likely due to error compensation—hPF potentials are in general softer than two-body ones, therefore it is expected that hPF simulations result in softer density profiles than the (excessively sharp) MARTINI one.

### 3.4 Effect of σ on Molecular Assemblies

**Figure 6:**
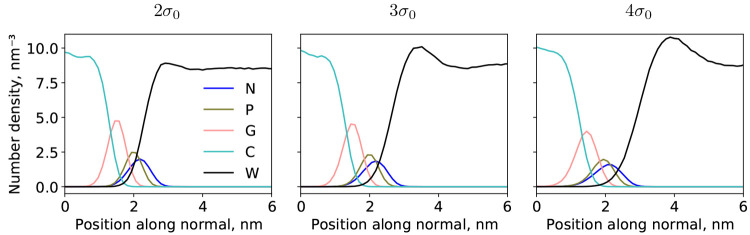
Symmetrized partial density profiles for HhPF simulations of solvated DPPC membranes at 323 K (DPPC5 system, see Table 1) for different values of the coarse-graining parameter σ. In each case, an equilibration time of at least 20 ns was allowed before sampling for at least 80 ns.

We have shown that at the matching coarse-graining, σ = σ_0_, the new framework reproduces well the bilayer structures of underlying particle–particle simulations. To further assess the effect of the coarse-graining parameter σ in the HhPF scheme, we report density profiles of the DPPC bilayer system in Figure 6. As expected, the smoother potential resulting from higher σ has a smearing effect on the membrane and the resulting density profiles. It is evident that the increased σ yields stronger phase separation between the hydrophobic lipid carbon tails and the solvent. This is in accordance with previously reported results for phospholipid bilayers in the hPF-MD model, wherein increasing the grid spacing (effectively increasing the range of the nonbonded field interaction) results in a more severe carbon–water segregation and a narrowing of the density profiles.^11^ The more extreme cases of σ = 3σ_0_ and σ = 4σ_0_ show artificial buildups of solvent outside the boundaries of the bilayer, resulting from the strong carbon–water interaction *through* the bilayer head groups, because of the increased effective range of the -interactions.

In large part, the deformed density profile at larger σ-values is a result of the carbon–water  interaction. The appropriate value used at the σ = σ_0_ level of coarse-graining is too extreme for the quadrupled σ case. If extensive simulations at the new level of coarse-graining are desired, a reoptimization of the -matrix is warranted, using, e.g., our previously reported Bayesian optimization scheme.^40^ However, note carefully that even though the large-σ bilayer structure is distorted with regards to the all-atom reference simulation, the lamellar phase is still stable and retains its overall organization.

### 3.5 Self-Assembly of Lipid Bilayers

Phospholipids spontaneously aggregate into bilayer structures in aqueous environments, and self-assembly of model phospholipids has been observed in numerous coarse-grained and all-atom MD simulations.^55−64^

The spontaneous assemblage of biological membranes is currently difficult to observe experimentally, however uni- or multilamellar membrane structures *at equilibrium* have been thoroughly studied for decades.^65−69^ As such, verification of molecular force fields and simulation procedures is normally done by comparison with equilibrium properties, e.g., easily accessible lateral electron density profiles across the resulting membrane.

The time scale of bilayer aggregation reported in CG-MD simulations is usually on the order of hundreds of nanoseconds.^55,59−61^ After a rapid initial phase of lipid–water separation, a proto-bilayer is formed containing aqueous pores. Closure of these pores then takes place in the tens to hundreds of nanoseconds regime. In all-atom simulations, the corresponding aggregation time is usually reported in the same hundreds of nanoseconds to microseconds range.^70,71^

**Figure 7:**
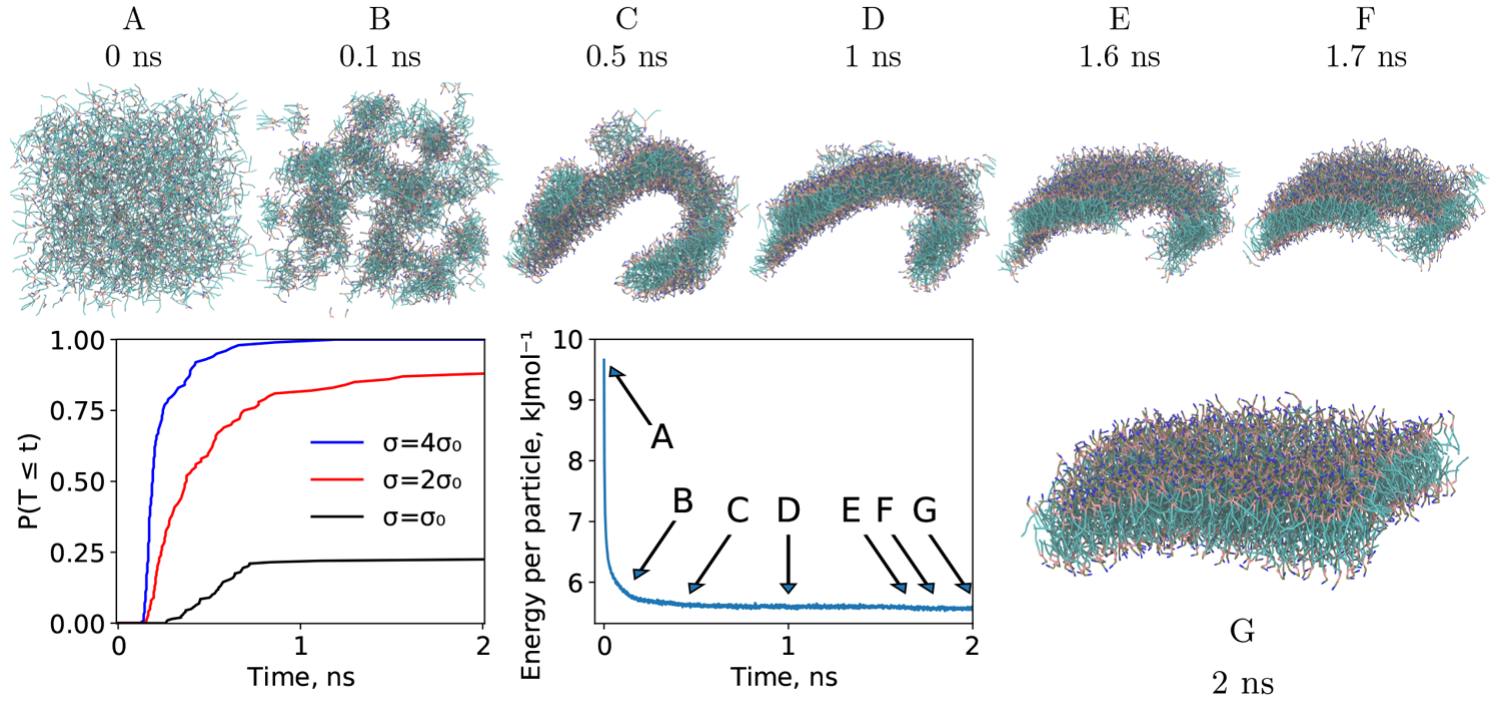
Total energy per particle and representative snapshots from a self-assembly simulation of a DPPC bilayer (DPPC5 system, see Table 1) under NVT conditions. Bottom-left: Cumulative probability distribution of time to reach a bilayer conformation, *T*, *F*_*T*_ = *P*(*T* ≤ *t*), for 400 test DPPC bilayer membrane simulations for different values of the coarse-graining parameter σ.

In the present formulation of the HhPF scheme, self-assembled bilayer structures appear much more rapidly than in corresponding CG simulations reported in the literature—in the subnanosecond regime. Figure 7 reports time to aggregate a perfectly symmetric bilayer from a randomized starting arrangement for select values of the coarse-graining parameter σ. We observe the tunable acceleration of the dynamics with varying σ. For the baseline σ = σ_0_, 22.5% of a trial of 200 test simulations ended up coalescing into a unilamellar structure within the first 2 ns. The corresponding ratio for 2σ_0_ and 4σ_0_ were 88% and 100%, respectively. Whenever immediate unilamellar aggregation does not occur, the system is stuck in a metastable proto-bilayer state which persists on the hundreds of nanoseconds time scale.

We may exploit this remarkably fast self-assembly procedure. As the soundness of the resulting structure is chiefly important, rapid structure aggregation is very beneficial. The organization arising from the spontaneously assembled bilayer with the transferred  interaction strengths depends on σ: Values closer to σ_0_ will yield better structures.

Since the desired structure is not always achieved within the first few nanoseconds in the high-resolution low-σ regime, we may utilize the capability of changing σ on the fly. A coarse-graining parameter of exactly σ_0_ yields the best fitting structure, but only instantaneous aggregation in about a quarter of trial simulations. Whereas the conformations obtained from 4σ_0_ simulations are not as good, self-assembly happens consistently. Utilizing the strengths of both approaches, we may start out simulations in a coarse representation, while rapidly decreasing σ over the first few nanoseconds. Tests of this scheme shows an approach that always results in immediate aggregation to a bilayer conformation of the best fit.

The speed-up of assembly dynamics as compared to literature hPF is dramatic, and represents the only major discrepancy between hPF and HhPF we encounter in the present work. Besides the explicit impact of the coarse-graining parameter σ, we attribute much of this speed-up to the choice of temperature control. hPF temperature control has traditionally been done by application of the Andersen thermostat, which has the direct advantage that no inter-CPU communication of local kinetic energy is necessary to calculate the instantaneous temperature. On the other hand, the Andersen coupling violates Galilean invariance and can eventually lead to unphysical disruption of transport properties,^72,73^ for example, significantly lowering self-diffusion of macromolecules, as demonstrated by, e.g., Basconi et al.^74^ This choice of temperature coupling therefore may hinder the inherently fast aggregation dynamics in hPF-MD by cooling translational degrees of freedom of supramolecular structures, frustrating e.g. micellar or vesicular fusion processes central in self-assembly events. The choice of a CSVR thermostat^39^ avoids the problematic aspects of the Andersen at the cost of slightly increased internode communication during thermostat application. Despite that, these additional computational costs are more than compensated by recovering the ultrafast aggregation dynamics expected in hPF models.

### 3.6 Self-Assembly of Nonlamellar Lipid Phases

PC type phospholipids are the most abundant lipids in biological membranes. In eukaryotic cells, these appear in large-scale cellular- or organelle-enclosing bilayer conformations. The bending rigidity of PC phospholipid aggregates hinders the formation of small-scale vesicles, on the contrary, facilitated by mixing with different lipids and sterols.

On the other hand, the poly-acylated bacterial Lipid A liposaccharide is characterized by a sufficient plasticity that enables its fast aggregation into regular vesicles, as inferred by dynamic light scattering experiment.^75^ A recent hPF model^41^ demonstrated how such an approach can be effective in studying both lamellar and nonlamellar phases of such complex lipids. In particular, they were able to predict the coexistence of micellar and vesicular structures of Lipid A just above the critical micellar concentration and suggested the (meta)stability of regular spherical vesicles formed by more than 600 lipids. Lipid A is thus an excellent test system to verify the ability of the HhPF approach toward the description of self-aggregation of complex charged systems.

**Figure 8:**
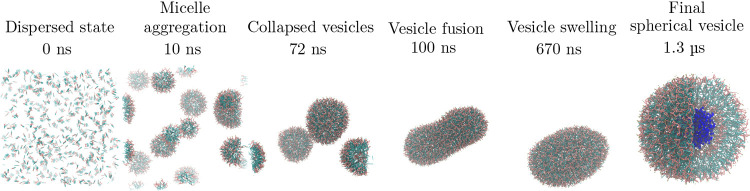
Sequence of snapshots showing the formation of a water-filled vesicle of Lipid A (LIPIDA system, see Table 1) under NVT conditions. The aggregation happens through early condensation into micelles, micellular fusion into collapsed vesicles, which further fuse into one larger unit that swells through water permeation across the vesicular wall. Solvent and counterions, present in the simulations, are omitted for clarity, except for the last snapshot which shows a (zoomed in) quarter cutout of the final vesicle with the enclosed water displayed in dark blue.

In our test, we start from 664 dispersed Lipid A/Ca^2+^ molecules in water. This number corresponds to the largest preconstituted vesicle studied in ref (41). During HhPF simulations, we observe a sudden phase separation between the water and the lipid phases. Within the first 100 ns, light-molecular weight micelles coalesce to form small collapsed vesicles that eventually fuse into a single unit. Across a further 1 μs, the vesicle swells by slow water permeation into the inner core, gradually acquiring a spherical shape. The final structure, reached after around 1.3 μs has an external radius of ∼15.5 nm and a thickness of ∼3.6 nm, in excellent agreement with all-atom^76^ and hPF simulations^41^ (Figure 8). The final self-assembled vesicle is formed by 202/442 Lipid A units in the inner/outer leaflet, respectively, strikingly close to the lipid partitioning (204/440) of the preassembled vesicle used in ref^41^.

#### 3.6.1 Aggregation of Charged Surfactants into Micelles

**Figure 9:**
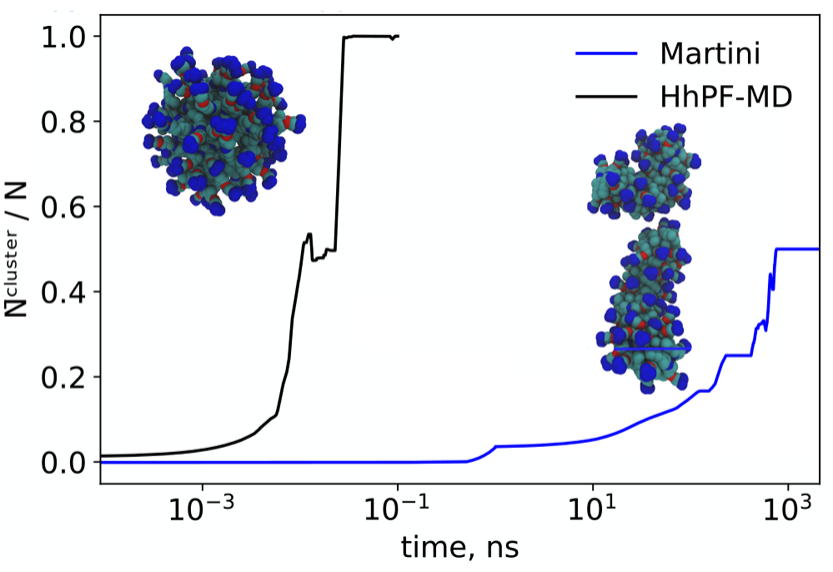
Clustering of AzoTMA into micelles, starting from random conformations (AZOTMA1 and AZOTMA2 systems, see Table 1) simulated under NVT conditions. Resultant structures for HhPF (left inset) and MARTINI (right inset) are shown as snapshots of the last frames of their respective simulations. A density-based spatial clustering of applications with noise clustering algorithm from sci-kit learn^78,79^ was used to identify and classify clusters. The resulting *N̅*/*N* was subsequently smoothed by a Savitzky–Golay filter^80^ with polynomial order unity.

The photosensitive surfactants 4-butyl-4-(3 trimethylammoniumpropoxy)-phenylazobenzene (AzoTMA) have been reported to form spherical micelles in aqueous solutions.^77^ However, due to the strong electrostatic repulsion between small micellar units, it remains challenging to observe the aggregation of AzoTMA into micelles from a dispersed state in coarse-grained MD simulations in the microsecond time scale. In Figure 9, we report the relative clustering size of AzoTMA micelles as a function of time obtained by performing HhPF or MARTINI simulations using the parameter sets in ref (44) (AZOTMA1 and AZOTMA2 systems, see Table 1). The MARTINI model fails to produce spherical micelles in the microsecond time-scale, yielding only smaller oblate aggregates. On the contrary, adopting the same coarse-grained mapping, HhPF captures the expected micellar structure in the nanosecond time scale, resulting in an aggregation acceleration of at least more than 3 orders of magnitude. The radius of the final micelle structure in the HhPF simulation is 3.2 nm, in excellent agreement with the experimental dynamic light scattering value of 3.1 nm ±0.6 nm.^77^ The lowered micelle fusion barrier likely results from the replacement of point-charge to smoothed charge interactions.

### 3.7 Polypeptide–lipid Membrane Interaction

**Figure 10:**
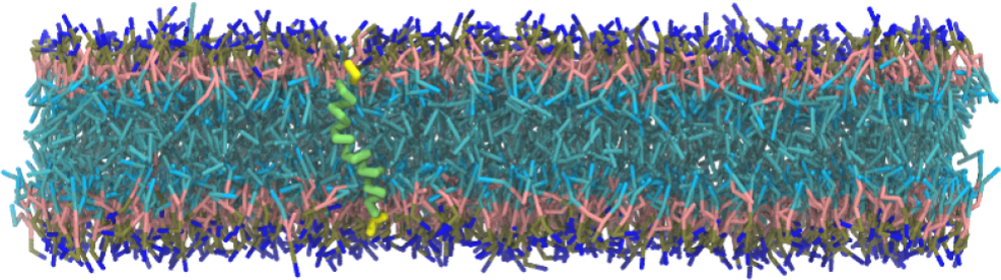
Snapshot of a 30 aa-long helical polypeptide (hydrophilic ends shown in yellow, hydrophobic core shown in green) embedded in a DOPC bilayer (PEPTIDE system, see Table 1) simulated under NVT conditions.

Additionally, we test the reliability of a recent hPF model for peptides^18^ within the HyMD implementation of the HhPF framework. Here we simulate a 30 residues-long helical polypeptide, where the last 3 amino acids on each end of the chain are hydrophilic, while the core region is hydrophobic, inserted in a DOPC membrane. The HhPF scheme is perfectly able to reproduce the peptide-bilayer interaction in agreement with previous hPF-MD simulations,^18^ with the peptide remaining embedded inside the lipid bilayer in a trans-membrane configuration and retaining its helical structure for the whole length of the simulation (Figure 10).

### 3.8 Computational Scaling

**Figure 11:**
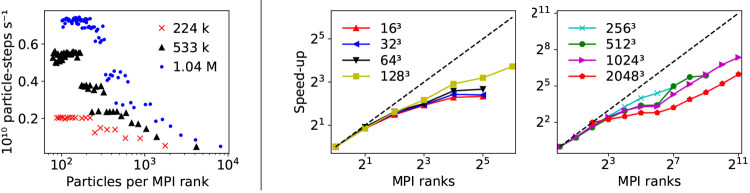
Left: Particle-steps per second for intramolecular bonds. Homopolymer test systems MELT1, MELT2, and MELT3 (see Table 1) are used, with 224,990 (red crosses), 533,310 (black triangles), and 1,041,620 (blue dots) particles, respectively. Right: Relative speed-up for the FFT-based intermolecular field force calculations for different FFT mesh sizes. Due to memory limitations, it was not possible to run the 2048^3^ grid points simulation for <4 MPI ranks; hence, the speed-up of the 2048^3^-mesh grid case is shown relative to 4 CPUs.

Figure 11 resumes the computational performance of this first release of the HyMD code. The inner rRESPA steps pertaining to the intramolecular bonded force calculation are inherently embarrassingly parallel (Figure 1). However, due to a non-negligible overhead, increasing the number of CPUs beyond a certain fraction of particles per MPI rank will not yield increased performance. As can be seen in Figure 11 (left), this ceiling is reached at approximately 200 particles per CPU. While this incurs a limit on the *strong scaling* behavior of the software, it is in reality inconsequential. In absence of particle–field interactions, the peak performance of the bonded terms—on a modest 5000 MPI ranks—exceeds 13 μs sampled per day for the test system containing one million particles (MELT3, see Table 1).

The reciprocal space code performance is presented on the right-hand side of Figure 11. Depending on the mesh grid size used in the 3D FFTs, we find limited scaling up to 2048 CPUs. Near-optimal scaling behavior is found only in the smallest tested MPI configurations. The 2D *pencil grid* domain decomposition used in the PMESH^35^ library theoretically scales to *N*^2^ CPUs for 3D Fourier transforms of linear dimension *N*.^81^ In fact, the PMESH backend PFFT found near-ideal scaling for 256^3^ total grid points up to approximately 16 000 MPI ranks.^31^ Despite limitations in the efficiency of the present version of the HyMD code, we are nonetheless able to reach a sampling time of approximately 2.0 μs per day for systems containing one million particles using a stable rRESPA configuration. While this is not an optimal performance, it is already enough to probe the physics of interesting molecular soft systems in a coarse-grained representation. The significant discrepancy between the current performance of HyMD and the underlying PMESH libraries indicate great potential for further optimization of the code, which will be the aim of its next release.

The main objective of the present work is to validate the HhPF formalism on realistic molecular systems, also providing a computational platform to exploit the method. For this first released version of the HyMD software, comparatively less attention has been devoted to the optimization of the performance. We fully expect the efficiency to drastically increase in the coming months as we enter the next phase of development.

## 4 Conclusion

In this work, we present the validation and full implementation of the recently proposed Hamiltonian formalism of the hybrid particle-field model framework. We verify that HhPF reproduces microcanonical dynamics equations in the presence of molecular moieties, in particular adopting a rRESPA multiple-time step algorithm splitting the intramolecular and field interactions. We find that the necessary time interval of the outer loop is solely dependent on the density spread σ.

We demonstrate how the efficiency of the inherently accelerated HhPF dynamics can be harnessed to rapidly achieve near-equilibrated self-assembled structures. Following this formulation, the level of coarse-graining may be changed on the fly to yield better results during a sampling phase. Alternatively, the model may be exchanged altogether for a particle–particle model at the same coarse grained level.

Simulations of a range of different surfactants yield unilamellar and nonlamellar structures corresponding well to those of literature hPF simulations, of higher accuracy approaches, or of experiments. We propose how avoiding kinetic traps in self-assembly may be done by increasing the coarse-graining parameter σ or through a simple *simulated annealing* strategy involving scheduled raising and lowering of σ on the fly.

The new formalism is compatible with existing formulations of the hybrid particle-field scheme, facilitating the mutual exchange of optimized parameter sets, regardless of the original approach used during optimization. The agreement between hPF and HhPF models depends on the grid spacing used in the canonical hPF and the σ parameter in HhPF. The use of symbolic differentiation renders the code agnostic with respect to the specific form of the energy functional, easily opening up to the implementation any other modeling of the hPF interactions.^16,82^ Thanks to the possibility of systematically controlling the numerical error associated to grid operations, yielding correct microcanonical mechanics, the HhPF implementation in HyMD promises to be an excellent tool for cross-validation and benchmarking of different density functional-based simulation methods. Moreover, the reciprocal-space implementation of noncovalent interactions provides an excellent setup toward interfacing with particle-based codes in a multiresolution manner.^83^

The current version of HyMD code has been fully validated for constant volume simulations only. In fact, recently schemes for hybrid particle-field simulations at constant pressure have been proposed.^53,84,85^ This much needed addition to the theory opens up the formalism to a range of important applications for which constant volume conditions are not the most appropriate. Here, we anticipate that the development of constant pressure HhPF equations and their implementation into the HyMD code has been recently achieved and will be the topics of a forthcoming publication.

While this early software implementation is fast enough to be useful, it nevertheless suffers from inefficiencies when compared to the computational scaling of the underlying FFT library. The HyMD code is currently an order of magnitude off of the reported scaling behavior of PFFT;^31^ however, we fully expect to match that CPU scaling in the long term.

Finally, HyMD is the first released module of the Hylleraas Software Platform (HSP) https://gitlab.com/hylleraasplatform/hylleraas. With the aim to cover the research activities at the Hylleraas Centre, HSP is developed into a unified framework for the study of molecular systems and their interaction with external forces and fields. The Python-based framework couples various in-house and external chemistry codes and allows for the study of systems spanning a wide range of size and time scales. In addition to the focus on research, the platform is also developed to become a tool in support of teaching activities in chemistry and related disciplines at all undergraduate levels. Within the HSP, we aim to progressively include a variety of multiscale tools into HyMD, covering the molecular/mesoscale dimensionalities, including dissipative particle dynamics, Brownian dynamics, and Monte Carlo-based methods.

## Data Availability

HylleraasMD is provided with a LGPLv3 open source software license and is accessible at our GitHub Web site https://github.com/Cascella-Group-UiO/HyMD.

## Supporting Information Available

HhPF interaction energy parameters  for DPPC, DOPC, Lipid A, AzoTMA, and the model Alanine peptide; coarse-grained mapping for AzoTMA; DPPC5 (see Table 1) momentum conservation; and an outline of the intramolecular bonded force field terms used in HyMD (PDF)
