# Supplementary material for: HylleraasMD: A Domain Decomposition-Based Hybrid Particle-Field Software for Multiscale Simulations of Soft Matter
Source: J Chem Theory Comput. 2023 May 2;19(10):2939–52. doi: 10.1021/acs.jctc.3c00134 (PMC10210244; doi:10.1021/acs.jctc.3c00134)
Supplement: Supplementary file 1 — ct3c00134_si_001.pdf [file ct3c00134_si_001.pdf]

Supporting information for: HylleraasMD: A Domain Decomposition-Based Hybrid Particle-Field Software for Multi-Scale Simulations of Soft Matter

Morten Ledum,[†] Samiran Sen,[†] Xinmeng Li,[†] Manuel Carrer,[†] Yu Feng,[‡] Michele Cascella,[*,†] and Sigbjørn Løland Bore[*,¶]

† *Department of Chemistry, and Hylleraas Centre for Quantum Molecular Sciences, University of Oslo, PO Box 1033 Blindern, 0315 Oslo, Norway*

‡ *Berkeley Center for Cosmological Physics and Department of Physics, University of California, Berkeley, CA 94720, United States*

¶ *Department of Chemistry and Biochemistry, University of California San Diego, La Jolla, California 92093, United States*

E-mail: michele.cascella@kjemi.uio.no; sbore@ucsd.edu

Contents

S1 Origin and justification of the HhPF method 3

S2 $\tilde{\chi}$-interaction parameters used in simulations 4

S3 Coarse-graining of AzoTMA 5

S4 Energy and momentum (per particle) conservation for the DPPC test system 6

S5 Intramolecular bonds 7

Two-body stretching bonds 7

Three-body angular bonds 7

Four-body torsional bonds 8

References **8**

S1 Origin and justification of the HhPF method

The hybrid-particle field (hPF) method originated in the field of polymer physics.[1,2] In such systems, excluded volume effects strongly influence the conformational dynamics, rendering it important to be dealt with carefully. However, in the presence of such effects, the probability distribution of the chain conformation cannot be calculated exactly. Therefore we have to resort to approximations, one of which is the mean field approach where single chains interact with the density field created by the other moieties in the system.

One of the early works on the hPF method was a single chain in mean field developed within a Monte Carlo formalism by Müller et al,[3] which was applied to homopolymers and block copolymers. Milano and Kawakatsu[4] built upon this, proposing a molecular dynamics self-consistent field methodology (MD-SCF) using a coarse-grained representation that was employed to study systems comprising polymers, lipids and surfactants. In this line of development, Bore and Cascella[5] recently proposed an energy-conserving hPF approach with a fundamentally different construction of the density fields, which we have elaborated on in the main text.

Like most standard molecular simulation theories, the Hamiltonian of the system is composed of a term with bonded interactions and another with non-bonded interactions. The bonded terms are detailed in S5 while the non-bonded terms are not based on particle-particle interactions but instead we formulate an external field using density functional theory, with which particles interact.

A major advantage of this method is the computational efficiency owed to the reduced number of interactions one has to deal with in the calculation of potential and forces.

S2 $\tilde{\chi}$-interaction parameters used in simulations

Table S1: $\tilde{\chi}$-matrix parameters used. All self-interaction parameters, $\tilde{\chi}_{ii}$, are zero, and $\tilde{\chi}_{ij} = \tilde{\chi}_{ji}$ in general. DPPC parameters are taken from Ledum et al.,[40] Lipid A parameters are taken from De Nicola et al.,[41] DOPC and peptide parameters are taken from Bore et al.[18] and Ledum et al.[40] AzoTMA parameters are based on the Martini mapping originally introduced in ref.[44]

DPPC

	W	C	G	D	P
N	-3.77	13.56	1.97	7.20	-9.34
P	-1.51	14.72	8.04	11.70	
D	23.25	0.0	6.30		
G	4.53	10.47			
C	42.24				

Lipid A

	L	P	G	C	N
W	0.0	-3.6	4.5	33.75	0.0
N	0.0	-7.2	0.0	13.25	
C	13.25	20.0	8.3		
G	4.5	4.5			
P	0.0				

DOPC and peptide

	W	C	G	D	P	ALA	PHI
N	-2.46	14.39	4.92	7.2	-3.4	14.39	-2.46
PHI	0.0	35.0	9.07	23.25	-1.27	0.0	
ALA	35.0	0.0	14.61	0.0	12.52		
P	-1.27	12.52	8.45	11.7			
D	23.25	0.0	6.3				
G	9.07	14.61					
C	35.0						

AzoTMA

	C	S	O	M	N	W	CL
T	4.40	6.40	11.40	0.0	17.0	30.70	34.0
CL	28.29	21.50	13.50	34.0	11.50	-1.59	
W	26.29	19.50	14.50	30.71	7.90		
N	10.79	4.0	2.0	17.0			
M	4.40	6.40	11.4				
O	6.99	2.0					
S	2.0						

S3 Coarse-graining of AzoTMA

The coarse grained mapping used for the AzoTMA system is presented in Figure S1.

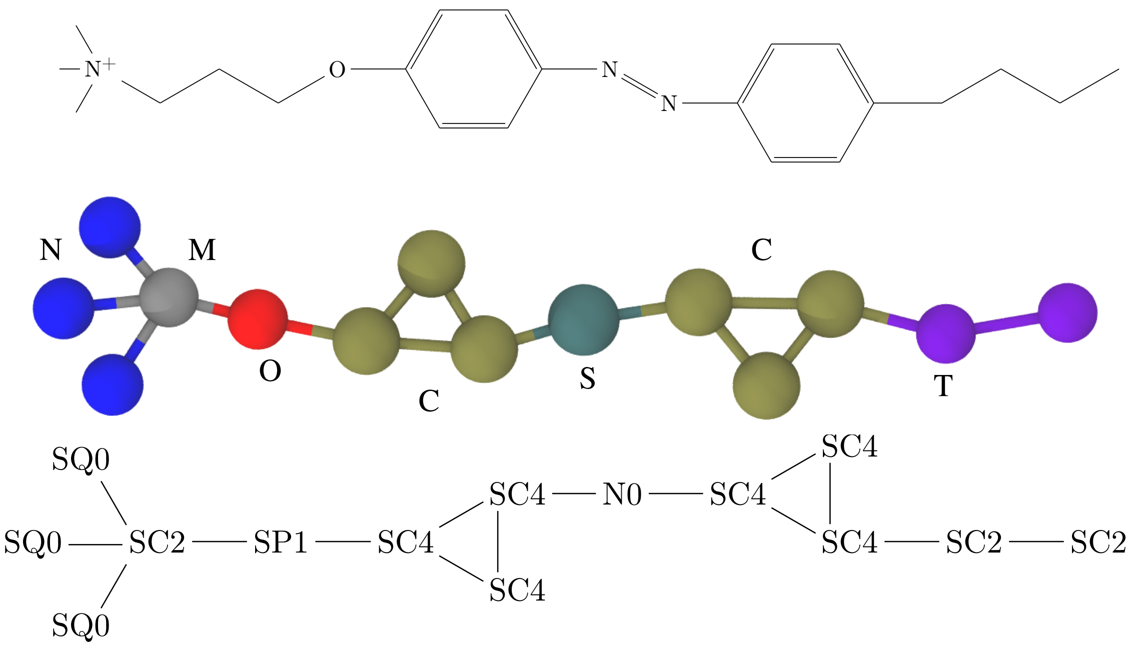

Figure S1: Atomistic representation of AzoTMA (top), coarse-grained mapping and bead names (middle), and Martini bead type specification (bottom). The counter-ion CL and solvent water W beads are not shown. The original Martini coarse-grained mapping is taken from ref.[44]

S4 Energy and momentum (per particle) conservation for the DPPC test system

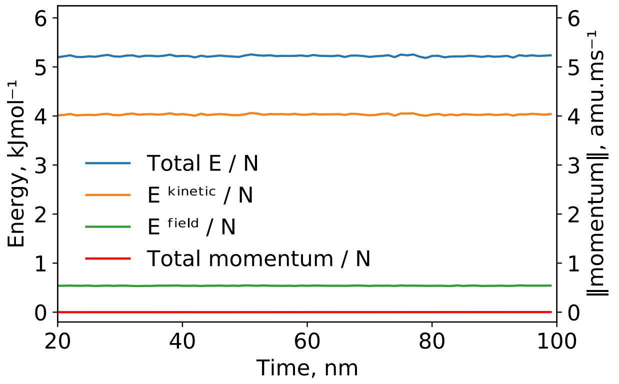

Figure S2: Energy and momentum (per particle) conservation for the DPPC5 test system, see Table 1 in the main text.

S5 Intramolecular bonds

Two-body stretching bonds

Stretching bonds in HyMD are represented by harmonic spring potentials,

$$V(r) = \frac{k}{2}(r - r_0)^2, \tag{1}$$

where $|\mathbf{r}| \equiv r$ is the inter-particle distance, k is a force constant of dimension energy, and r_0 is the equilibrium bond distance. The force is calculated as

$$F_{i\to j}(\mathbf{r}) = k(r - r_0)\frac{\mathbf{r}}{r}. \tag{2}$$

Three-body angular bonds

Three-body bonds in HyMD are harmonic in the angle θ,

$$V(\theta) = \frac{k}{2}(\theta - \theta_0)^2, \tag{3}$$

where θ_0 is an equilibrium angle and k is a constant of unit energy. From the three particles involved (labelled a, b, and c), the angle of the bond is computed through the law of cosines as

$$\theta = \cos^{-1}\left(\frac{\mathbf{r}_a \cdot \mathbf{r}_c}{r_a r_c}\right), \tag{4}$$

where $\mathbf{r}_a$ is the vector from b pointing to a, with $\mathbf{r}_c$ correspondingly pointing from b to c. The force on a and c are calculated by

$$\mathbf{F}_a = -\frac{\mathrm{d}V(\theta)}{\mathrm{d}r_a}\frac{\mathrm{d}r_a}{\mathrm{d}\mathbf{r}_a} \tag{5}$$

and

$$\mathbf{F}_c = -\frac{\mathrm{d}V(\theta)}{\mathrm{d}r_c}\frac{\mathrm{d}r_c}{\mathrm{d}\mathbf{r}_c}. \tag{6}$$

The force on b is found by the combination

$$\mathbf{F}_b = -\mathbf{F}_a - \mathbf{F}_c. \tag{7}$$

Four-body torsional bonds

Dihedral bonds in HyMD are cosine series potentials between four particles (a, b, c, and d), depending on the angle between the a-b-c and b-c-d planes. The potential is defined as

$$V(\phi) = \sum_{n=0}^{4} c_n(1 + \cos(n\phi - \phi_n)), \tag{8}$$

for appropriate values of the coefficients c_n (units of energy) and the phases ϕ_n. The force felt by each particle participating in the dihedral angle is given by

$$\mathbf{F}_i = -\frac{dV(\phi)}{d\phi}\frac{d\phi}{d\mathbf{r}_i} \tag{9}$$

The explicit calculation of the second derivative can be found in the literature.[6]

References

(1) Kawakatsu, T. *Statistical physics of polymers: an introduction*; Springer Science & Business Media, 2004.

(2) Rubinstein, M.; Colby, R. H., et al. *Polymer physics*; Oxford university press New York, 2003; Vol. 23.

(3) Daoulas, K. C.; Müller, M. Single chain in mean field simulations: Quasi-instantaneous field approximation and quantitative comparison with Monte Carlo simulations. *J. Chem. Phys.* **2006**, *125*, 184904.

(4) Milano, G.; Kawakatsu, T. Hybrid particle-field molecular dynamics simulations for dense polymer systems. *J. Chem. Phys.* **2009**, *130*, 214106.

(5) Bore, S. L.; Cascella, M. Hamiltonian and alias-free hybrid particle–field molecular dynamics. *J. Chem. Phys.* **2020**, *153*, 094106.

(6) Blondel, A.; Karplus, M. New formulation for derivatives of torsion angles and improper torsion angles in molecular mechanics: Elimination of singularities. *J. Comput. Chem.* **1996**, *17*, 1132–1141.
